# Feasibility of Outpatient High-Dose Methotrexate Infusions in Pediatric Patients With B-Lineage Acute Lymphoblastic Leukemia

**Published:** 2018-05-01

**Authors:** Joy L. Bartholomew, Hongying Dai, Keith J. August, Robin E. Ryan, Kristin A. Stegenga

**Affiliations:** Children’s Mercy Hospital Kansas City, Kansas City, Missouri

## Abstract

High-dose methotrexate (MTX) given in four hospitalizations during interim maintenance for high-risk pediatric B-lineage acute lymphocytic leukemia significantly improves survival but increases resource utilization. Children remain hospitalized for intravenous hydration and blood or urine monitoring until MTX clearance parameters are reached. Improved supportive care, extended infusion center hours, and pediatric home health expertise afford alternatives to prolonged hospital admissions, potentially offering quality, cost-effective approaches that positively impact the delivery of care.

Acute lymphoblastic leukemia (ALL), the most common form of pediatric leukemia, accounts for 26% of all cancers occurring in children from birth to 14 years of age (Ward, DeSantis, Robbins, Kohler, & Jemal, 2014). Of the 3,000 new pediatric cases arising each year in the United States (Ries et al., 1999), nearly half are considered high risk (HR). High-risk disease, defined at diagnostic presentation, is age 10 years or above and/or initial white blood cell (WBC) count greater than or equal to 50,000/mL. Additional cytogenetic findings and the level of residual disease on days 8 and/or 29 during induction therapy may modify a prior risk classification from standard to high (Hunger et al., 2013). Regardless of when the HR classification was determined, the therapy is similar.

Standard therapy for high-risk B-cell lineage ALL includes five treatment phases administered over 2.5 to 3.5 years, depending on the gender. The third phase of therapy is interim maintenance, which consists of four 24-hour continuous infusions of high-dose methotrexate (HD MTX) every 14 days (Larsen et al., 2011). Treatment with HD MTX in interim maintenance became the standard of care for high-risk pre–B-cell ALL after recent clinical trial results demonstrated significantly improved event-free survival compared to standard therapy. Methotrexate is an antifolate that has been used in a variety of pediatric cancers at varying doses since the 1950s. Although this is an old medication with a well-established toxicity profile, HD MTX infusions are routinely administered in the hospital for a variety of reasons, including intravenous (IV) hydration, drug level and urine monitoring, as well as historical practice.

Chemotherapy is the most frequent cause for pediatric oncology hospitalizations (Russell et al., 2014). In 2011, the US Agency for Healthcare Research and Quality estimated the direct medical cost of oncology hospitalizations to be $31 billion (American Cancer Society, 2018). High-dose methotrexate hospital stays are most often 3 to 4 days in length, but in cases of prolonged excretion, they can be 7 days or longer. These hospitalizations pose a significant burden to the child, family, and society, likely adding millions of dollars to overall US health-care expenses. These additional chemotherapy admissions also burden the health-care physical infrastructure by putting further pressure on an already stretched inpatient bed availability; this may be especially true for smaller institutions and free-standing children’s hospitals. Bed space limitations may lead to delays in treatment, a problem that has been associated with decreased survival outcomes.

The success of short MTX infusions, such as for the treatment of osteosarcoma, and home postchemotherapy hydration has been well established (Lippert et al., 2017; Mahadeo et al., 2010). This pilot aimed to assess the feasibility of a 24-hour continuous home MTX infusion in adherence to best care practices established by an MTX clearance algorithm, while ensuring high-level supportive care in a cost-effective manner for children with ALL.

Innovative methods that deliver high-quality, evidence-based care are key to controlling cost. Improved supportive care, extended infusion center hours, and pediatric home health (HH) expertise have afforded alternatives to hospital admissions. Such alternatives that can offer quality, cost-effective approaches that may positively impact the delivery of care and subject satisfaction warrant investigation.

## METHODS

**Sample Size**

The free-standing pediatric institution in this pilot study averages 180 new oncology patients each year, of which 25% have leukemia. Of these patients with leukemia, 30% have high-risk B-cell ALL. A sample size of 5 reflects 37% of patients who are diagnostically eligible. The anticipated HH costs and our current inpatient charges were estimated and statistically compared to determine if a sample size of 5 would identify a significance in cost benefits. Based on these considerations, a total of 5 subjects was determined to be an adequate sample size.

**Subject Selection**

The hematology/oncology department used an admission calendar to track scheduled hospitalizations. Chemotherapy admissions are typically listed 2 to 4 weeks prior to the anticipated appointment. After the study was reviewed and approved by the institutional review board, potential subjects were identified by viewing this list and subsequently approached prior to the scheduled chemotherapy admission. Seven subjects were recruited, and two denied participation.

**Subjects**

A convenience sample of 5 subjects aged 3 to 16 years with de novo high-risk ALL completed 6 outpatient infusions and 14 inpatient infusions of MTX at 5 g/m² at a free-standing pediatric academic hospital between March and September 2015. Three subjects had government insurance, and two had commercial insurance. One of the subjects received two infusions in the outpatient setting due to reported significant improvement of quality of life for the family when therapy was delivered in the home. Although this second outpatient therapy was not funded by the study, the outpatient data were approved for inclusion. All subjects resided within one of the 33 counties serviced by the institution’s HH company and included both urban and rural areas. Subjects had Lansky scores greater than 70, were fluent in English, had no known ascites or effusions, and had no history of renal insufficiency. Consents were obtained prior to the start of HD MTX therapy.

**Study Design**

**Figure 1:**
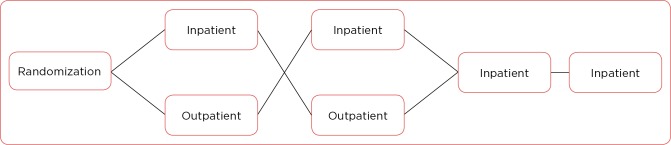
Study design.

This study was a randomized, crossover design with subjects assigned to either their first HD MTX infusion administered as an outpatient or as an inpatient. The second HD MTX infusion was infused in the alternate venue (Figure 1).

**Process**

Subjects presented to the clinic for evaluation; labs and physical exams were confirmed to be adequate for therapy. All subjects had sulfa medications, proton pump inhibitors, and nonsteroidal anti-inflammatory drugs held the day of the MTX infusion. All subjects at this institution had home antiemetics for use as needed; an ample supply was verified prior to the start of the MTX infusion.

Those subjects who received inpatient therapy were admitted to the hospital from the clinic, which is the institution’s standard practice. The HD MTX was infused over 24 hours, followed by alkalinized IV fluids (IVF) post MTX completion. Methotrexate levels were drawn at hours 24, 42, and 48, and daily electrolytes were also obtained as per the current standard of care. Additional labs, IVF, and leucovorin changes were implemented as needed and subjects remained inpatient until MTX excretion, all based on an algorithm that is considered standard of care.

Subjects scheduled to receive outpatient therapy arrived at the clinic at 10:00 am to ensure that the exam, labs, and, if needed, lumbar puncture could be completed in time for a 2:00 pm MTX infusion start. This timing ensured that lab draws with completed results were available during daytime hours in case adjustments were needed. Once deemed ready for therapy by physical exam and laboratory results, subjects were met in the clinic by the HH company and connected to the outpatient MTX infusion pump; subjects were discharged from the clinic with the HD MTX infusing. At the completion of the HD MTX infusion at home, an HH nurse went to the subject’s home, disconnected the MTX, obtained an hour 24 MTX level and electrolytes, and initiated alkalinized IVF. IV fluids continued at home until adequate clearance was determined by MTX levels. Electrolytes and MTX levels were obtained at hours 42 and 48 in the subject’s home by HH. All blood specimens were processed by the institution’s laboratory. If the MTX or creatinine levels exceeded the expected values, IVF and/or the frequency of leucovorin administration were increased based on the current MTX standard-of-care algorithm. Lab results were followed by an advanced practice nurse (APN) and were reported to the family and the HH. During these calls to the family, the urine pH was reported. Any potential adjustments were coordinated by the APN.

All hospital admissions and outpatient infusions were scheduled on Mondays or Tuesdays to ensure that comprehensive services were available. Scheduled admissions for routine chemotherapy do not routinely occur on the weekend at this institution, and therefore this did not alter standard scheduling for either inpatient or outpatient delivery. During the day, between 8:00 am and 5:00 pm, lab results were monitored by the principal project APN for outpatient service and by the inpatient APN chemotherapy service for those who were hospitalized. Any unanticipated subject care needs or parental concerns in the outpatient cohort were to be evaluated in clinic. Questions or concerns that arose outside office hours were to follow the same procedure currently in place for speaking to the on-call hematology/oncology APN. Pump issues were to be directed to HH.

An action plan based on the current standard of care for monitoring MTX in pediatrics was implemented in both venues. If the urine pH was consistently too high or too low, the alkalization of the IVF would be adjusted by the APN. In the home setting, the need for IVF modification would be reported to the HH company and delivered to the home by the nurse with the next scheduled lab draw, or sooner if needed. The algorithm advises that if the 24-hour MTX level is greater than or equal to 150 µM or the creatinine is greater than 125% baseline, a repeat level should be drawn at hour 36. If the MTX level is greater than 3 µM and the creatinine is still greater than 125%, the hydration should be increased to 200 mL/m²/hr; if this occurred in the outpatient venue, the HH nurse would return to the home to adjust the IVF rate if the family was uncomfortable with adjusting the pump themselves. Subsequent MTX and creatinine levels that were not responding to the increased hydration per the algorithm would have prompted admission in the outpatient setting.

**Data Collection**

Each subject was assigned a consecutive number and the data were recorded accordingly. Objective data were collected from clinic arrival time until the MTX level reached less than 0.4 µM, or if there was a history of delayed excretion, less than or equal to 0.1 µM via electronic medical record. Data included the start time of MTX infusion, frequency of antiemetic use, urine pH, serum creatinine and MTX levels, IVF rates per m², changes in IVF additives, dosing and frequency of leucovorin, and the incidence of vomiting and mucositis. Methotrexate levels were recorded at all standard time points (hours 24, 42, and 48) and as needed until MTX clearance.

Objective data specific to the outpatient setting were collected on a standardized form completed by the subject and caregiver. This included the incidence of vomiting and mucositis, frequency of antiemetic use, and urine pH. To determine urine pH, litmus paper and a color chart indicating pH was provided. Education in this process and subject or caregiver understanding was confirmed prior to the start of the HD MTX infusion. Subjects were phoned with the results of each MTX level and serum electrolytes and the home urine pH testing was then reviewed.

The billable charges for each venue were obtained through the institution’s electronic accounting system and did not include any insurance adjustments. Charges included clinic visits, chemotherapy, IVF and IV fluid additives such as potassium chloride and sodium acetate, infusion pump rental, inpatient hospital bed, and medical provider and HH nursing. Charges for labs, antiemetics, and leucovorin were not collected, and actual subject billing statements were not reviewed.

Out-of-pocket expenses associated with both inpatient and home infusions were recorded by the caregiver. A standardized form was provided and utilized for both the inpatient and home settings. Examples of out-of-pocket expenses included child care and meal purchases.

**Data Analyses**

Continuous variables were summarized using mean and standard deviation, while categorical variables were summarized by count and percentage. Univariate analysis was performed to compare each variable between inpatient and outpatient administration using paired t-tests. Methotrexate levels, creatinine levels, antiemetic usage, and incidence of vomiting were individually evaluated intrasubject. Profile plots were generated. Statistical analyses were performed using SAS 9.4. Statistical significance was claimed at the 95% confidence level.

## RESULTS

All six home infusions were completed without incident. Specifically, there were no equipment or infusion issues, no subsequent clinic or emergency room visits, and no hospitalizations due to therapy intolerance or adverse events. Worksheets on which subjects or caregivers tracked the incidence of antiemetic use, vomiting, and urine pH were all completed and returned to the project staff. No IVF rate, IVF additive, or leucovorin dosing changes were implemented. There were also no occurrences of mucositis or urine pH levels that fell below 7 in either setting.

There was a significant difference between billable care charges as an inpatient compared to the outpatient MTX administration (*p* = .004). Inpatient charges ranged from $10,921 to $44,533, and outpatient charges from $1,981 to $3,197 (Table 1). The medication cost for MTX comprised an average of 7% of the inpatient charges and 25% of the outpatient charges. Hospital floor charges were the highest billable cost for inpatient stays, and skilled nursing care was the highest cost in the outpatient setting.

**Figure 2:**
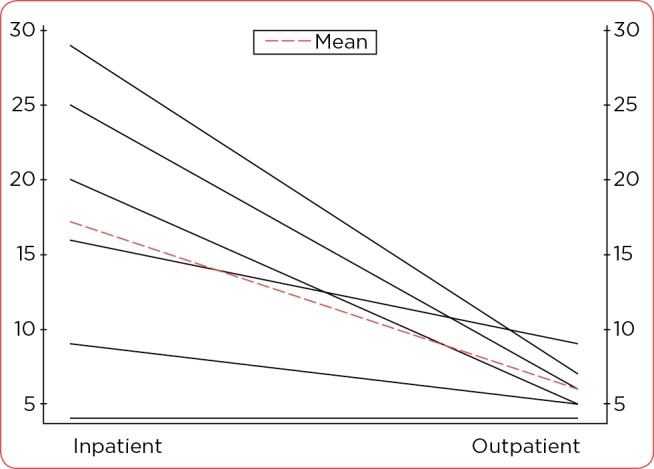
Paired profiles for antiemetic use.

Time until MTX clearance was not significantly affected by venue (*p* = .18). However, a trend toward more rapid clearance of MTX was seen when comparing the inpatient and outpatient means (74 hours and 52 hours, respectively), which suggests that significance may have been detected in a larger sample size. Since MTX levels were often checked more than once daily in the hospital, an analysis was performed adjusting the inpatient times to 24-hour intervals; there was no resulting change (p = .16).

Changes in creatinine during each cycle (*p* = .48; mean: outpatient 0.47, inpatient 0.50) and incidence of emetic episodes (p = .46; mean: outpatient 1, inpatient 2) were not compromised in the home setting. Whereas the number of emetic episodes was similar, there was a significant decrease in the use of antiemetics in the outpatient compared to the inpatient setting (p = .02). The mean for antiemetic administrations in the inpatient setting was 17.2 (range 4–29), and for the outpatient setting it was 6 (range 4–9). Only one subject used the same number of antiemetic doses in both venues; all others used less (Figure 2).

**Table 1:**
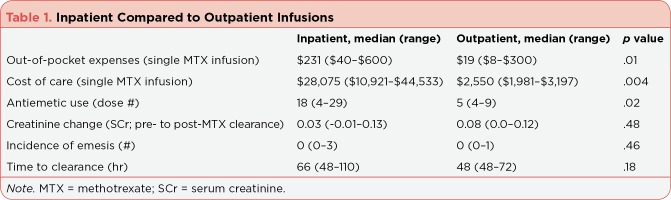
Inpatient Compared to Outpatient Infusions

## DISCUSSION

With so much emphasis on curing childhood cancer, little attention is paid to improving the delivery of care and reducing costs. We investigated the feasibility of home administration of HD MTX as an alternative to prolonged inpatient hospitalizations, with a goal of reducing cost and assessing the delivery of care in an outpatient venue. We found a 10-fold increase in the charges associated with inpatient administration of HD MTX compared to outpatient infusion, with the actual cost of the MTX medication itself contributing very little. The study population was small; therefore, demonstrating significance is noteworthy.

Antiemetic use was significantly reduced in the outpatient setting. The reason for this is likely multifactorial. High doses of MTX are moderately emetogenic, and all subjects may not require scheduled administration of antiemetics as is typically practiced during inpatient chemotherapy admissions. Also, there is likely a psychologic benefit to being in the home environment that could lead to a reduced need for antiemetics.

There were no infusion pump errors, inadequate HH availability, or any other event that prompted outpatients to subsequently be admitted. We did not have any adverse events associated with home administration of HD MTX such as elevated creatinine or poor MTX clearance. In fact, time to clearance in this small group of subjects was less for outpatient administration when compared to inpatient administration, although this finding did not reach statistical significance.

This study had several limitations, the most recognized being the small subject population, although this sample size is common when determining feasibility. Additionally, this study occurred at a single center utilizing an institutional HH care company well versed in pediatric expertise, and such services may not be available to other centers. Finally, home administration of antiemetics and incidence of emesis documentation was presumed to be accurate but was unable to be objectively verified.

This feasibility study indicated that home administration of HD MTX is safe and cost effective in pediatric subjects with B-lineage ALL and is more tolerable as evidenced by a significant decrease in antiemetic use. Further investigation in larger studies of outpatient HD MTX is warranted to prove equivalence and to statistically determine if clearance and quality of life is improved in children with ALL. These results also suggest a need to consider outpatient options for other childhood cancer therapies.

**Acknowledgment**

This article is supported by the Kansas City Area Life Science Institute, Kansas City, Missouri.

## References

[1] American Cancer Society. (2018). Economic impact of cancer.. https://www.cancer.org/cancer/cancer-basics/economic-impact-of-cancer.html.

[2] Hunger Stephen P, Loh Mignon L, Whitlock James A, Winick Naomi J, Carroll William L, Devidas Meenakshi, Raetz Elizabeth A (2013). Children’s Oncology Group’s 2013 blueprint for research: acute lymphoblastic leukemia.. *Pediatric blood & cancer*.

[3] Larsen E C, Salzer W L, Devidas M, Nachman J B, Raetz E A, Loh M L, Carroll W L (2011). Comparison of high-dose methotrexate (HD-MTX) with Capizzi methotrexate plus asparaginase (C-MTX/ASNase) in children and young adults with high-risk acute lymphoblastic leukemia (HR-ALL): A report from the Children’s Oncology Group Study AALL0232 [Abstract 3].. *Journal of Clinical Oncology*.

[4] Lippert M, Semmens S, Tacey L, Rent T, Defoe K, Bucsis M, Shykula T, Crysdale J, Lewis V, Strother D, Lafay-Cousin L (2017). The Hospital at Home program: no place like home.. *Current oncology (Toronto, Ont.)*.

[5] Mahadeo Kris M, Santizo Ruth, Baker Lindsay, Curry Joan O'Hanlon, Gorlick Richard, Levy Adam S (2010). Ambulatory high-dose methotrexate administration among pediatric osteosarcoma patients in an urban, underserved setting is feasible, safe, and cost-effective.. *Pediatric blood & cancer*.

[6] Ries L A G, Smith M A, Gurney J G, Linet M, Tamra T, Young J L, Bunin G R (1999). *Cancer incidence and survival among children and adolescents: United States SEER Program 1975–1995.*.

[7] Russell H V, Okcu M F, Kamdar K, Shah M D, Kim E, Swint J M, Ho V (2014). Algorithm for analysis of administrative pediatric cancer hospitalization data according to indication for admission.. *BioMed Central Medical Informatics and Decision Making*.

[8] Ward E, DeSantis C, Robbins A, Kohler B, Jemal A (2014). Childhood and adolescent cancer statistics, 2014.. *CA: A Cancer Journal for Clinicians*.

